# Developing an Artificial Intelligence-Based Pediatric and Adolescent Migraine Diagnostic Model

**DOI:** 10.7759/cureus.44415

**Published:** 2023-08-30

**Authors:** Shiori Sasaki, Masahito Katsuki, Junko Kawahara, Chinami Yamagishi, Akihito Koh, Shin Kawamura, Kenta Kashiwagi, Takashi Ikeda, Tetsuya Goto, Kazuma Kaneko, Naomichi Wada, Fuminori Yamagishi

**Affiliations:** 1 Department of Neurosurgery, Japanese Red Cross Suwa Hospital, Suwa, JPN; 2 Department of Neurosurgery, Itoigawa General Hospital, Itoigawa, JPN; 3 Department of Health Promotion, Itoigawa City, Itoigawa, JPN; 4 Department of Neurology, Itoigawa General Hospital, Itoigawa, JPN; 5 Department of Neurology, Japanese Red Cross Suwa Hospital, Suwa, JPN; 6 Department of Surgery, Itoigawa General Hospital, Itoigawa, JPN

**Keywords:** telemedicine, smartphone application, pediatric migraine, machine learning, coronavirus disease 2019 (covid-19)

## Abstract

Introduction

Misdiagnosis of pediatric and adolescent migraine is a significant problem. The first artificial intelligence (AI)-based pediatric migraine diagnosis model was made utilizing a database of questionnaires obtained from a previous epidemiological study, the Itoigawa Benizuwaigani Study.

Methods

The AI-based headache diagnosis model was created based on the internal validation based on a retrospective investigation of 909 patients (636 training dataset for model development and 273 test dataset for internal validation) aged six to 17 years diagnosed based on the International Classification of Headache Disorders 3rd edition. The diagnostic performance of the AI model was evaluated.

Results

The dataset included 234/909 (25.7%) pediatric or adolescent patients with migraine. The mean age was 11.3 (standard deviation 3.17) years. The model’s accuracy, sensitivity (recall), specificity, precision, and F-values for the test dataset were 94.5%, 88.7%, 96.5%, 90.0%, and 89.4%, respectively.

Conclusions

The AI model exhibited high diagnostic performance for pediatric and adolescent migraine. It holds great potential as a powerful tool for diagnosing these conditions, especially when secondary headaches are ruled out. Nonetheless, further data collection and external validation are necessary to enhance the model’s performance and ensure its applicability in real-world settings.

## Introduction

Facts about headache treatment practice

Headache is one of the common neurological diseases. Migraine is a public health problem [1-10], and they are described in the International Classification of Headache Disorders 3rd edition (ICHD-3). The prevalence of migraine is not low at 0.9-9.5% [8,9,11-18]. The recognition of migraine’s economic and social impacts on productivity is becoming more apparent [17,19]. Currently, there is widespread utilization of novel migraine drugs like calcitonin gene-related peptide (CGRP)-related drugs, such as galcanezumab, fremanezumab, erenumab, and serotonin 1F receptor agonists like lasmiditan. While awareness of migraine among patients and healthcare providers is gradually improving and new drugs are being introduced, there is still an unmet medical need for migraine care: 89.8% had never used preventative medicine for headaches, and 36.5% expressed hesitancy in consulting doctors. Both individuals experiencing less frequent and more frequent headache attacks reported significant disability and interictal burden, and impacts on productivity [20] and quality of life [9,21]. Presumably, most headache and migraine sufferers manage their pain using over-the-counter (OTC) medicines [22,23]. Moreover, when individuals with headaches seek medical consultation, doctors often rely solely on neuroimaging to rule out organic or urgent conditions, resulting in inadequate diagnosis and treatment for detailed primary headaches. Even in cases of diagnosing primary headaches, clinicians often lack the necessary understanding of suitable treatments, leading to patient dissatisfaction [22,24]. Improper utilization of OTC medications and inadequate medical resources for headache management can contribute to the development of chronic migraine and medication-overuse headache (MOH) [25,26]. Namely, appropriate preventative drugs can be used [27-30], but migraine patients do not consult doctors, resulting in the aggravation of migraine. This significant public health issue about migraine could be averted by promoting headache awareness and the appropriate use of acute and preventative medication. Especially in addition to educating the public, it is essential to be able to properly diagnose migraine [10,21].

Migraine among children and adolescents

Indeed, migraine is a significant concern among children and adolescents as well. Numerous school-based surveys with a narrow focus on specific age groups have been conducted to assess the prevalence of migraine among children and adolescents [31-45]. According to recent school-based studies worldwide, the prevalence of migraine among children and adolescents ranges from 1.7% to 11.0% [22]. In Japan, the prevalence of migraine among elementary and junior high school students was 3.5%-11.3% [12,16,46,47]. Those among Japanese high school students were 15.6% [48]. They may miss school or have difficulty with their studies [12]. Also, MOH can develop in children and adolescents [12,39-43,45,49,50]. Just like adults, children and adolescents also require accurate diagnosis and appropriate treatment for migraine. This includes the use of prophylactic medications and acute care medications as needed to manage their migraine episodes effectively. Proper medical attention and care are crucial in ensuring the well-being of children suffering from migraines [22]. The diagnosis of migraine should be the first step in order to initiate treatment properly.

Diagnosing migraines in children poses challenges due to the intricacies of gathering their medical history. Conducting a clinical interview incorporating relevant questions, eliciting comprehensive responses, and encouraging children to maintain a headache diary can be demanding [51,52]. Furthermore, during the developmental phase, it is plausible for migraines and tension-type headache (TTH) to co-occur in pediatric patients. There is a potential for these headache types to alternate or transition from one to the other over time during the follow-up period [53]. Alternative methods, such as drawing pictures, are being investigated as potential diagnostic tools for headaches in children who may have difficulty verbally expressing their symptoms [51,52]. Still, they are difficult for non-specialists in headache, such as general pediatricians, school teachers, school nurses, and parents, to tackle diagnosing pediatric migraine.

Artificial intelligence and migraine diagnosis

Automated headache diagnosis systems employing artificial intelligence (AI) are becoming increasingly prevalent. These systems offer the potential to address misdiagnosis by non-specialists, as they save time during a medical interview while simultaneously enhancing diagnostic accuracy [10,24,54-60]. There is still a shortage of headache specialists and inappropriate treatment of migraine headaches by physicians with no knowledge of headaches. With headache diagnosis AI, even non-specialists can correctly diagnose migraine in a short time with less burden. Patients would then be better able to enjoy the correct treatment. However, there have been no pediatric and adolescent migraine diagnosis models using AI.

This study tested the hypothesis that migraine can be diagnosed by objective measures alone. By creating this AI model, even a layperson can determine whether the child has migraine or not based on objective findings alone, due to the challenges associated with expressing symptoms through language by pediatric and adolescent patients. This way, more children who have not yet seen a doctor will see a medical professional when they realize they have migraines, allowing for early detection and early treatment. With this in mind, the primary objective was to create an AI-based pediatric migraine diagnosis model that relies on items easily comprehensible to individuals around children. To this end, AI was developed and its diagnostic accuracy, such as sensitivity and specificity, was verified.

## Materials and methods

Study design

The AI diagnosis model was developed through a retrospective analysis of 909 questionnaire sheets from children with headaches. Among these, 636 sheets were utilized for training the model, and 273 were used as a test dataset to evaluate its performance. The 907 of the 909 questionnaire sheets were collected as part of a school-based online epidemiological study on children’s and adolescents’ headaches conducted in the year 2022 [12]. The questionnaire sheet consisted of the following 15 items: age, biological sex, family history of headache, past history of, apart from headache, motion sickness, light-headedness, photophobia, phonophobia, dizziness, disease duration of headache, how many days per month headache occurs in these three months or no headaches, characteristics as 1) unilateral location, 2) pulsating quality, 3) moderate or severe pain intensity, 4) aggravation by or causing avoidance of routine physical activity, 5) nausea and/or vomiting, 6) abdominal pain, 7) photophobia, 8) phonophobia, and 9) osmophobia, the headache duration, what acute medication you use, how many days per month you use the acute medication, use of prophylactic medication for headache, and what prophylactic medication you use. We also asked about the disturbance to daily life as an experience of absence from school, being patient during activity, early leaving, depression, difficulty attending class, difficulty in after-school activities, and consulting doctors. We then asked whether the coronavirus disease 2019 (COVID-19) pandemic increased or decreased the headache frequency and its reason as a free description. We finally asked migraine respondents about 25 potential triggers for headache: fatigue, weather change, rainy day, hot day, lack of sleep, after getting up, staying home, cloudy day, stress, windy day, menstruation, smartphones or video games, exercise, excess of sleep, holiday, after school, snowy day, allergy and hay fever, sunny day, sports, wearing a mask, going to school, fighting or argument, hunger, and after eating. This predetermined list of triggers included behavioral, dietary, environmental, and hormonal factors [61]. The questionnaire sheets asked as nominal variables except for age. The questionnaire sheets were made for the epidemiological survey named Itoigawa Benizuwaigani Study [12], and made by two headache specialists and three neurologists. Amid the COVID-19 pandemic, schools in Itoigawa city sometimes had to close. Since 2021, each student received a tablet for remote learning. The Niigata Prefectural Board of Education initiated this interactive approach in 2019. From April to August 2022, an online survey was conducted five times with reminders, involving 14 elementary, four junior high, and three high schools. Students and a parent jointly completed the questionnaire on their tablets or devices. All public school students in Itoigawa city were included, provided they responded to all items. Those declining participation were excluded. Blank submissions were not accepted to ensure data validity.

The migraine and non-migraine headache diagnosis was based on ICHD-3 criteria, without seeing the patients by doctors. The rest of the two adolescent or pediatric patients’ questionnaire sheets of the 909 sheets were collected in the headache outpatient of the Japanese Red Cross Suwa Hospital from April to July, 2023. Although the headache outpatient is basically for adults, children and adolescents sometimes visit the headache outpatient, and we examined the two patients during this period. Using the questionnaire and medical examination, three skilled neurologists diagnosed whether the two patients had migraine based on the ICHD-3, after sufficient discussion.

**Figure 1:**
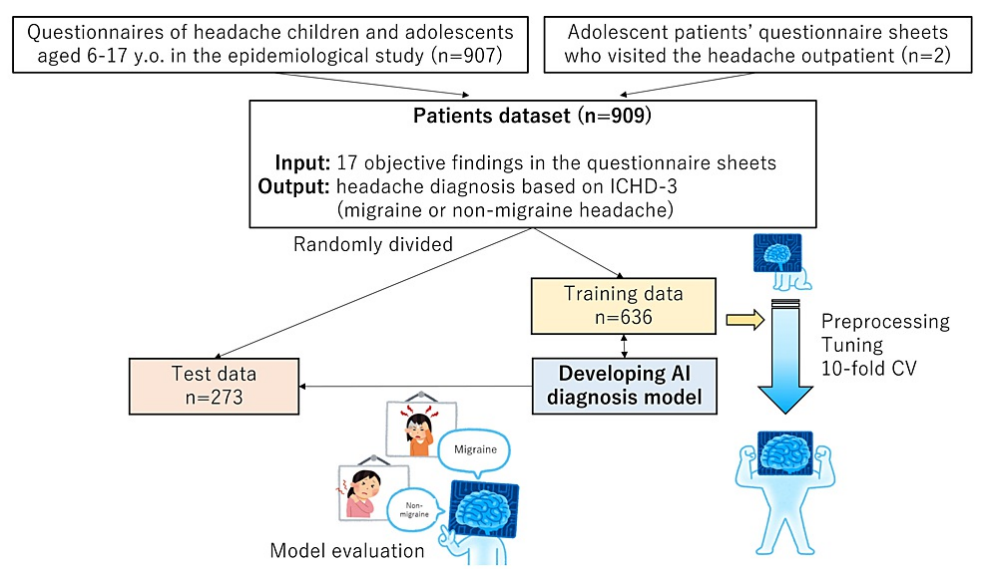
Study design An AI diagnosis model was developed based on a retrospective investigation of 909 headache patients diagnosed based on the ICHD-3. The data of the 909 patients were randomly divided into 636 training and 273 test datasets. The AI diagnosis model was developed using the training dataset with preprocessing, hyperparameter tuning, and 10-fold CV. Then its performance was tested using the test dataset. AI, artificial intelligence; CV, cross-validation; ICHD-3, International Classification of Headache Disorders, 3rd edition

The AI diagnosis model was constructed using the questionnaire datasets of 636 patients, which underwent preprocessing, hyperparameter tuning, and cross-validation. Subsequently, its performance was evaluated using the test dataset of 273 patients. It’s important to note that the model’s development was solely based on the 636 training dataset, and the 273 test dataset was not used during the model’s production to avoid any bias in its evaluation (Figure 1).

Developing an AI diagnosis model

The 909 questionnaire sheets originally included these items; age, biological sex, family history of headache, past history of, apart from headache, motion sickness, lightheadedness, photophobia, tinnitus or phonophobia, dizziness, disease duration of headache, how many days per month headache occurs in these three months or no headaches, characteristics as 1) unilateral location, 2) pulsating quality, 3) moderate or severe pain intensity, 4) aggravation by or causing avoidance of routine physical activity, 5) nausea and/or vomiting, 6) abdominal pain, 7) photophobia, 8) phonophobia, 9) osmophobia, and 10) vertigo, the headache duration, what acute medication you use, how many days per month you use the acute medication, use of prophylactic medication for headache, and what prophylactic medication you use. The questionnaire sheets also asked about disturbances to daily life as an experience of absence from school, being patient during activity, early leaving (resting in the nurse’s office), depression, difficulty attending class, difficulty in after-school activities, and consulting doctors.

Of these items in the questionnaire sheets, the AI-based model used 17 objective items and predicted migraine or non-migraine headache diagnosis. These 17 items were determined to be objectively understandable by parents, teachers, and others, even if the pediatric patient cannot express their headache’s characteristics verbally; age, biological sex, family history of headache, past history of, apart from headache, motion sickness, lightheadedness, photophobia, tinnitus or phonophobia, dizziness or vertigo, headache characteristics as aggravation by or causing avoidance of routine physical activity, nausea and/or vomiting, abdominal pain (stomachache), photophobia, phonophobia, and osmophobia, the number of days missed from school, the experience of missing school and resting in the nurse’s office.

PyCaret (https://pycaret.readthedocs.io/en/latest/index.html) was used to create the AI-based diagnosis model because it easily performs preprocessing, comparison of algorithms, and hyperparameter tuning. The general process was the same as in previous reports [10,60]. After putting 909 patients’ dataset into the PyCaret on Python notebook, PyCaret randomly divided 909 patients into training data of 636 patients and test data of 273 patients (7:3 ratio) by “get_data” and “setup” commands. The model production is only based on the 636 training dataset, and the performance was tested using the 273 dataset. For preprocessing, z-normalization for numerical variables was performed. Using the 636 training dataset, PyCaret made several predictive models with 10-fold internal cross-validation. The algorithm with the largest c-statistics (area under the curve of the receiver operating characteristic curve; AUC of ROC) was chosen after model comparison by the “compare_models” command among 14 AI models, including boosting method. The c-statistics were the averages of the 10-fold cross-validation. Hyperparameter tuning was then performed to maximize AUC by “create_model,” “tune_model,” and “finalize_model” commands after choosing one of the best models described above. Randomized search cross-validation was applied during hyperparameter tuning with 10 iterations. Finally, the 273 test dataset, which was still untouched, was predicted using the final tuned model. The accuracy, sensitivity (recall), specificity, precision, F-value, and c-statistics were used to evaluate the model’s performance. SHapley Additive exPlanations (SHAP) values [62] were used to understand why the AI outputs the patients’ diagnosis (https://shap.readthedocs.io/en/latest/#).

No external validation was performed at this time. However, for future external validation, a calibration curve was created for the model and performed calibration [63] by refitting based on logistic regression. The calibration was performed with “sklearn.calibration.CalibratedClassifierCV” commands for PyCaret.

The accuracy, sensitivity (recall), specificity, precision, F-values, and c-statistics were used to evaluate the model’s performance. The F-value is a harmonic mean of recall and precision. The F-value is used as an overall indicator of the trade-off relationship between recall and precision. Kappa index [64] and Matthews correlation coefficient [65] were also calculated.

Statistical analysis

Variables with normal distribution are expressed as mean (standard deviation), while those with a non-normal distribution are expressed as median (interquartile range). Mann-Whitney U test and Fisher exact test were used for statistical comparison. The evaluation indices of the AI model were assessed using the migraine diagnoses based on the ICHD-3 criteria as the reference standard (ground truth). SPSS 28.0.0 (IBM Corp., Armonk, NY, USA), scikit-learn 1.2.2, SHAP 0.42.1, Python 3.9.0, PyCaret 3.0.0, and Matplotlib 3.5.1 were used.

Ethical aspects

Itoigawa General Hospital Ethics Committee approved this study (approval number 2021-22). There were no names or other personally identifying information in the 907 questionnaire sheets obtained from the online anonymous survey. For the two Suwa Red Cross Hospital patients, informed consent for this study was obtained in writing. In this study, personal information was not identified, and anonymized information was used. All procedures were carried out following the Helsinki Declaration. This study design followed the Transparent Reporting of a multivariable prediction model for Individual Prognosis Or Diagnosis (TRIPOD) statement [66] and the guideline on developing and reporting machine learning predictive models in biomedical research [67].

## Results

Patient characteristics

Table 1 summarizes the diagnoses for the 636 patients in the training dataset and the 273 patients in the test dataset.

**Table 1:** Patient characteristics

	Training data	Test data
Number of cases	636	273
Mean age (standard deviation)	11.26 (3.17)	11.35 (3.16)
Biological sex (%Female)	39.9%	40.0%
Class 1; Migraine	167 (26.3%)	71 (26.0%)
Class 2; Non-migraine headache	469 (73.7%)	202 (74.0%)

Approximately 26% of the pediatric patients were diagnosed with migraine, while the remaining patients had non-migraine headaches, as per the criteria of the ICHD-3. The mean age of the patients was around 11 years, and approximately 40% of them were female. No statistically significant differences were observed in the baseline characteristics between the training and test datasets.

Developing an AI diagnosis model and its performance

Using the 636-training dataset, PyCaret revealed that the Extremely Randomized Trees [68] had the largest c-statistics of 0.9944 among the several algorithms (Table 2). Hyperparameter tuning was performed to optimize AUC; the learning results and hyperparameter tuning results are shown in Tables 3, 4.

**Table 2:** Model comparison AUC, area under the curve

	Model	Accuracy	c-statistics (AUC)	Recall	Precision	F1 value	Kappa	Matthews Correlation Coefficient	Training time
et	Extra Trees Classifier	0.967	0.9944	0.9518	0.9307	0.939	0.9164	0.9185	0.546
rf	Random Forest Classifier	0.9638	0.9922	0.946	0.9229	0.9329	0.9082	0.9096	0.532
xgboost	Extreme Gradient Boosting	0.9575	0.9898	0.9224	0.9205	0.9193	0.8906	0.8925	0.212
lightgbm	Light Gradient Boosting Machine	0.956	0.9911	0.9342	0.9074	0.9191	0.8889	0.8904	0.149
dt	Decision Tree Classifier	0.9387	0.9213	0.8853	0.8847	0.8826	0.8412	0.8433	0.207
gbc	Gradient Boosting Classifier	0.9387	0.9849	0.9099	0.8693	0.8868	0.8449	0.8474	0.294
lr	Logistic Regression	0.8789	0.9383	0.7243	0.8044	0.7583	0.678	0.6825	0.601
ridge	Ridge Classifier	0.8774	0	0.7243	0.8008	0.756	0.6747	0.6796	0.141
lda	Linear Discriminant Analysis	0.8773	0.9422	0.736	0.7938	0.7598	0.6778	0.6817	0.177
svm	SVM - Linear Kernel	0.8663	0	0.6761	0.8009	0.7251	0.6382	0.6483	0.104
ada	Ada Boost Classifier	0.8663	0.9329	0.6651	0.8051	0.7247	0.6375	0.6452	0.274
knn	K Neighbors Classifier	0.8649	0.9379	0.5772	0.8675	0.6877	0.6073	0.6307	0.259
dummy	Dummy Classifier	0.7375	0.5	0	0	0	0	0	0.114
qda	Quadratic Discriminant Analysis	0.5694	0.5852	0.5143	0.3733	0.3391	0.0825	0.1196	0.115
nb	Naive Bayes	0.5233	0.9067	0.9941	0.3593	0.5268	0.227	0.3231	0.192

**Table 3:** Learning results AUC, area under the curve; MCC, Matthews correlation coefficient

Fold	Accuracy	AUC	Recall	Prec.	F1	Kappa	MCC
0	0.9062	0.9837	0.8235	0.8235	0.8235	0.7597	0.7597
1	0.9375	0.9787	0.8235	0.9333	0.875	0.8336	0.8365
2	0.8906	0.97	0.7059	0.8571	0.7742	0.7029	0.7087
3	0.9062	0.9675	0.7647	0.8667	0.8125	0.7503	0.7529
4	0.875	0.9299	0.8235	0.7368	0.7778	0.6912	0.6933
5	0.8438	0.9574	0.5882	0.7692	0.6667	0.567	0.5757
6	0.8413	0.9066	0.7647	0.6842	0.7222	0.6116	0.6134
7	0.9048	0.9707	0.75	0.8571	0.8	0.7379	0.7407
8	0.8413	0.9668	0.5	0.8	0.6154	0.522	0.5449
9	0.8571	0.9774	0.5	0.8889	0.64	0.5594	0.5955
Mean	0.8804	0.9609	0.7044	0.8217	0.7507	0.6736	0.6821
Std	0.032	0.023	0.1221	0.0713	0.0817	0.0977	0.0903

**Table 4:** Hyperparameters of the model

bootstrap	FALSE
ccp_alpha	0
class_weight	None
criterion	gini
max_depth	None
max_features	sqrt
max_leaf_nodes	None
max_samples	None
min_impurity_decrease	0
min_samples_leaf	1
min_samples_split	2
min_weight_fraction_leaf	0
n_estimators	100
n_jobs	-1
oob_score	FALSE
random_state	0
verbose	0
warm_start	FALSE

**Figure 2:**
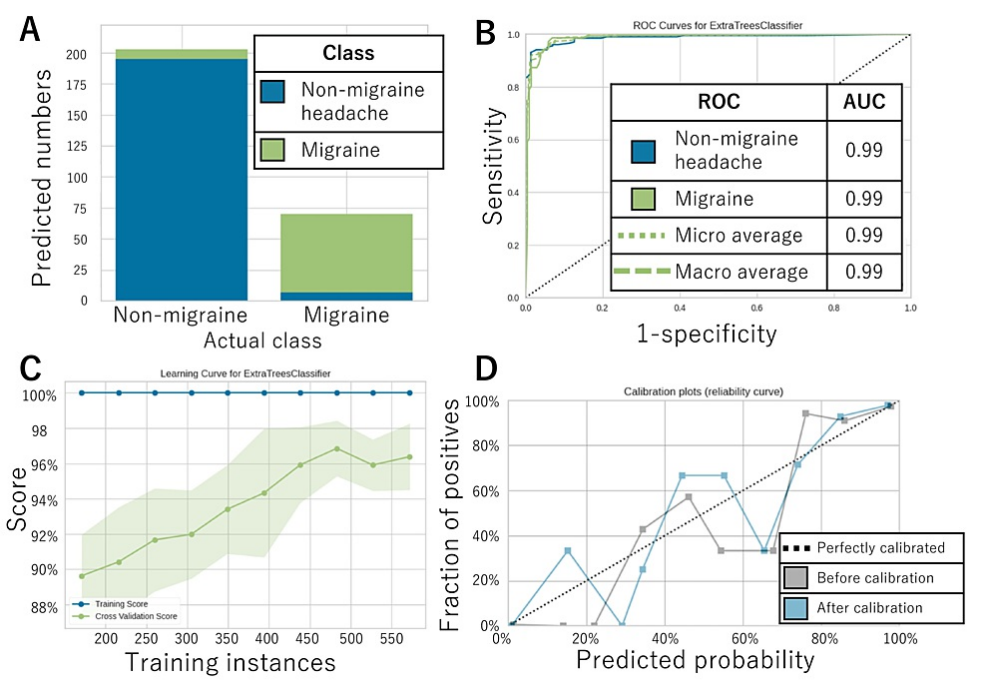
AI model’s performance The performance of the AI model for the test data is illustrated in A. The ROC curve for each class and its AUC are shown in B. The learning curve and calibration curve are shown in C and D, respectively. AI, artificial intelligence; AUC, area under the curve; ROC, receiver operating characteristic

The confusion matrix (Table 5) and error plot (Figure 2A) indicate the performance of the AI model for the 273-test dataset.

**Table 5:** Confusion matrix for the test dataset Accuracy 94.5%, Kappa index was 0.857 (95%CI 0.770-0.912), Matthews Correlation Coefficient 0.857. AI, artificial intelligence; ICHD-3, International Classification of Headache Disorders, 3rd edition

		Prediction by AI			Performance index			
		Class 1; Migraine	Class 2; Non-migraine headache	Total	Sensitivity (Recall)	Precision	Specificity	F-value
Ground truth based on ICHD-3	Class 1; Migraine	63	8	71	88.7%	90.0%	96.5%	89.4%
	Class 2; Non-migraine headache	7	195	202	96.5%	96.1%	88.7%	96.3%
	Total	70	203	273				

According to Table 2, the accuracy, sensitivity (recall), specificity, precision, and F-value for migraine were, respectively, 94.5%, 88.7%, 96.5%, 90.0%, and 89.4%. Figure 2B displays the ROC for migraine diagnosis along with its AUC, which was 0.99. The closer the AUC is to one day, the higher the discrimination performance, so a value of 0.99 is very high. Figures 2C and 2D, respectively, demonstrate the learning and calibration curves. In the learning curve, the cross-validation score became higher as the test data increased to 500 cases. On the other hand, it could have plateaued with further increase in sample size. Therefore, it is possible that a sample size of about 500 cases was sufficient for this modeling (Figure 2C). Even after calibration, the calibration curves were not well matched on the diagonal. This meant that the numbers output by the AI did not necessarily match the diagnostic probabilities. Caution should be exercised in interpreting the output numbers themselves (Figure 2D).

**Figure 3:**
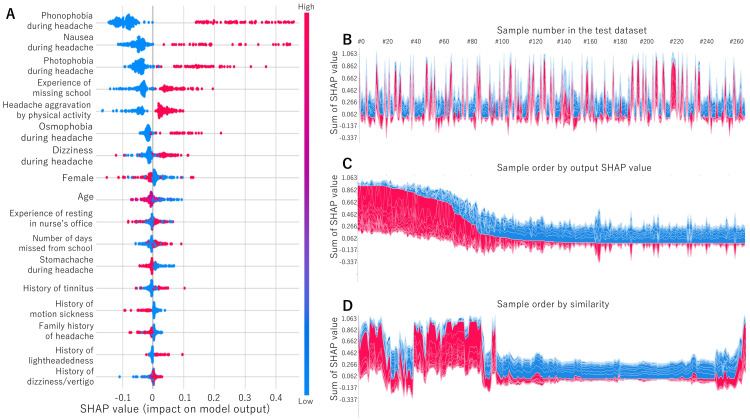
SHAP values The SHAP values are listed in order as A. The reason plot is shown in B (sample number order), C (SHAP value order), and D (order by similarity). SHAP, SHapley Additive exPlanations

Figure 3A displays the SHAP values. Phonophobia, nausea, photophobia during headache, the experience of missing school, headache aggravation by physical activity, osmophobia, dizziness during headache, female sex, age, the experience of resting in nurse’s office, number of days missed from school, stomachache during headache, history of tinnitus, motion sickness, family history of headache, history of lightheadedness, and dizziness/vertigo were important in that order. Figures 3B-3D show the reason plot for each sample based on the SHAP values. In some cases, the SHAP value was large, and the diagnostic reason could be identified, while in others, the SHAP value was small, and the diagnostic reason was difficult to identify (Figure 3C). There were some cases that were similar to each other (Figure 3D).

Fifteen patients were misdiagnosed. The reasons for the AI’s errors included questionnaires in which migraine was diagnosed based only on variables not used in this training, such as unilateral or pulsating, and questionnaires that had migraine characteristics but were secondary to headaches due to the common cold or trauma.

## Discussion

The AI-based headache diagnosis model was developed using the questionnaire dataset of 909 patients (636 training and 273 test datasets). The overall diagnostic performance using the test dataset was evaluated, and the diagnostic performance for migraine was high. This is the first study to report and discuss the effectiveness of an AI-based pediatric and adolescent migraine diagnostic model.

The burden of pediatric migraine

Pediatric and adolescent migraine is not simply related to disruption of schoolwork or absence from school due to migraine [69]. Pediatric and adolescent migraine and school refusal are closely related issues that can significantly influence social and intellectual development. Regular school attendance can be compromised when migraines become chronic or frequent, leading to difficulties in participating in educational activities.

It is estimated that about 2% of elementary, junior high, and high school students in Japan refuse to go to school [70,71]. School refusal, also known as school avoidance or school phobia, refers to a child’s consistent reluctance to attend school or the display of severe distress when faced with the idea of going to school. Unlike being driven by laziness or lack of motivation, this behavior is rooted in the genuine physical and psychological pain caused by migraines. Consequently, school refusal can profoundly impact a child’s academic performance and overall well-being, warranting careful attention and intervention to address both the migraines and the resulting school-related challenges [72]. Pediatric migraines may lead to school refusal, which, in turn, can negatively affect a child’s learning and social development. Frequent absences from school can result in academic challenges, increased workload backlogs, and reduced opportunities for social interactions with peers. Additionally, being unable to participate in daily school activities due to migraine attacks may make the child feel isolated and result in lower self-esteem. The child may also perceive themselves as different from their friends, further exacerbating feelings of loneliness. Addressing pediatric migraines and their impact on school attendance is crucial to supporting a child’s overall well-being and academic progress. Early diagnosis and appropriate management of migraines, coupled with providing understanding and support in the school environment, can significantly reduce the likelihood of school refusal and mitigate its negative consequences on the child’s life [70,71].

Migraine is not necessarily the only condition associated with school refusal [73]. However, it is necessary to pick up migraine children as much as possible and connect them appropriately to medical facilities, including headache specialists, in order to guide the patients to appropriate multidisciplinary collaborative treatment [74]. In pursuit of this objective, the AI-based diagnostic tool, which relies solely on objective items, promises to enable early detection and treatment of pediatric migraine cases. This could be a significant step forward in identifying and providing timely care for children who may be experiencing migraines.

Importance of early diagnosis of migraine

The notion that migraine is a progressive disease is being proposed [75,76]. It is estimated that episodic migraine progresses to chronic migraine at a rate of 2.5% per year and may be underestimated due to the arbitrary 15-day period according to the migraine criteria [77]. Key clinical features of migraine progression include increases in the number and intensity of attacks, autonomic disturbance, and allodynia, leading to chronic migraine over time [78]. Pathophysiologically, changes in hypothalamic activity, as one of the hypothesized generators of migraine [79], and diminished brainstem inhibitory process [80] have been estimated as the main progression mechanisms. The factors related to migraine progression are the high frequency of migraine attacks, medication overuse, comorbid pain syndromes, and obesity [75]. About 30% of chronic migraine is refractory to both preventive and acute treatment [22]. Although CGRP-related drugs have been reported to be effective in chronic migraine [81-85], their response rate is not comparable to that of episodic migraine.

Considering that migraine is a progressive disease and that migraine in childhood can continue into adulthood [86], the AI may be useful for early diagnosis of migraine without misdiagnosis.

Pediatric migraine diagnosis

Characteristics of migraine may differ between adults and children/adolescents. In children, migraine episodes usually extend beyond one hour and are associated with general neurovegetative symptoms, phonophobia, and often temporary neurological issues known as aura. Children commonly experience bilateral discomfort that is allodynic and accompanied by cranial autonomic signs, such as lacrimal discharge and rhinorrhea [87].

Early diagnosis and early intervention of migraine in childhood are important, given that migraine can be a progressive condition [77,79,88] and can interfere with schoolwork and school life [12]. The primary method for diagnosing primary headaches is the ICHD-3 criteria. However, when applied to the pediatric population, these criteria have shown certain limitations. Consequently, the ICHD-3 acknowledges specific characteristics of migraine in children, such as the relatively short duration of pain and whether it occurs unilaterally or bilaterally. Despite these considerations, there is only a moderate level of agreement between self-reported migraine attacks and those derived from the ICHD-3 criteria in adolescents and young children. This suggests that neither approach is flawless and may encompass overlapping features of the migraine condition [89].

Diagnosing migraine in pediatric patients can be challenging as they might have difficulty fully expressing their headache experiences verbally. Even with the ICHD-3 criteria, identifying migraine in children remains problematic, leading to potential underdiagnosis. The model was based on the questionnaire sheets diagnosed by ICHD-3 criteria. However, it demonstrates the ability to diagnose migraine using solely objective information accurately. By disseminating this model to non-specialists, such as families, schools, health departments, and general hospitals, appropriate referral of patients to headache specialists without overlooking any cases will be ensured. This could significantly improve the detection and management of pediatric migraines, ultimately benefiting young patients and their overall well-being.

Previous reports on AI-based diagnostic models

There is still no AI to diagnose migraine in children and adolescents. However, numerous models for diagnosing adult headaches using AI have been reported [59]. These diagnostic models should be assessed in the training and test datasets to prevent overfitting [67]. However, only seven models [10,54-58,60] have met this requirement, and they are summarized in Table 6.

**Table 6:** Previous reports on AI diagnosis for headache disorders Abbreviations: AI, artificial intelligence; LGBM, light gradient boosting machine; TACs, trigeminal autonomic cephalalgias; TTH, tension-type headache; MOH, medication-overuse headache. †, calculated in macro-average.

Author	Year	Output by the model	Methods	Variables	Training sample number	Test sample number	Validation sample number	%Migraine	Accuracy	Sensitivity (recall)	Specificity	Precision	F-value
Yin [55]	2015	2 class; Migraine or TTH	Case-based reasoning + Genetic algorithm	81	676	222	Not performed	76.10%	93.00%	97.02%	79.20%	93.14%	95.04%
Walters [54]	2016	2 class; Migraine or Other headache disorders	Logistic regression	4	887	942	Not performed	9.40%	92%	94%	92%	64%	93%
Vandewiele [56]	2018	3 class; Migraine, TTH, TACs	Decision tree	Not described	849	-	32	Not described	98%	98%	98%	Not described	Not described
Kwon [57]	2020	5 class; Migraine, TTH, TACs, Thunderclap headache, Epicranial headache	eXtreme Gradient Boosting,	75	1286	876	Not performed	68.49%	58.60%†	58.70%†	85.64%†	65.28%†	58.64%†
Cowan [58]	2022	2 class; Migraine or Other headache disorders	Decision tree	135	-	-	212	62%	92%	89%	97%	98%	93%
Katsuki [10]	2022	5 class; Migraine or MOH, TTH, TACs, Other primary headaches, Secondary headaches	Light gradient boosting machine	17	2800	1200	50	60.00%	90.00%	68.57%	95.00%	96.43%	88.08%
Katsuki [60]	2023	5 class; Migraine or MOH, TTH, TACs, Other primary headaches, Secondary headaches	Gradient boosting classifier	22	4240	1818	Not performed.	79.7%	93.7%†	40.6%†	48.5%†	88.7%†	43.5%†
This study	2023	2 class; Migraine or Other headache disorders	Extremely randomized trees	14	636	273	Not performed.	26.3%	94.5%	88.7%	96.5%	90.0%	89.4%

The model in this study exhibits similar performance levels as those reported in previous studies and demonstrates high accuracy in diagnosing migraine. However, given the variability of migraine prevalence among different cohorts, further investigation into AI diagnosis is necessary. Integrating radiomics data, along with descriptive information from medical questionnaires and examinations, holds the potential for enhancing diagnostic accuracy and uncovering novel medical insights [90]. While the model currently relies solely on questionnaire sheets, the inclusion of additional data from radiomics, clinical symptoms, and laboratory tests could be explored in future iterations to further improve its capabilities.

Smartphone applications and AI diagnosis

In recent times, the prevalence of smartphone applications for tracking headaches, so-called headache diaries, has significantly increased. These apps offer valuable contributions to headache diagnosis and prediction [91,92]. Headache diaries through smartphone applications are utilized as recording tools in pediatric headache research [93]. On the other hand, using smartphones is considered one of the most common triggers of migraine attacks in children [12]. Other possible negative effects on health using smartphones are also reported as text neck syndrome [94] and digital eye strain [95]. However, if these digital devices installed the AI diagnosis model, users could self-diagnose their migraine or parents and teachers could notice their children’s migraine, promoting access to appropriate information [19] and treatment options [58]. Additionally, as online medical care becomes more prevalent [96], by integrating a headache diary and diagnostic AI into a smartphone app, it becomes feasible to efficiently manage, assess, and treat headaches in a centralized manner. This innovative approach using a smartphone offers the potential for streamlined headache care, providing users with a comprehensive tool for monitoring, analysis, and personalized treatment right at their fingertips. In addition, smartphone applications can collect substantial real-world data that can be valuable for future research studies. By merging these applications with the AI model, the development of more accurate diagnostic models for the future becomes possible. It is crucial, however, to consider and address any potential detrimental impacts on children resulting from the use of smartphones in this context.

Limitations

This study has certain limitations that need to be addressed. The primary concern is the lack of generalizability. The dataset used for this study consists mainly of questionnaire sheets collected online in a rural Japanese city. It remains uncertain whether general clinics (such as general practitioners, family doctors, or general pediatrics) conducting initial headache examinations and clinical practice can achieve similar diagnostic performance when utilizing this AI in different settings, such as schools, homes, and school infirmaries where the presence of children’s headaches is first noticed. To establish the generalizability of the AI model, further confirmation of its diagnostic performance is required in a separate cohort study conducted in diverse contexts, including actual clinical settings in homes, schools, and general clinics. Additionally, it is essential to validate the model’s performance in other countries with varying headache prevalence rates, headache medical resources, and clinical environments.

Secondly, due to the development of this model mainly from questionnaire sheets without in-person consultation, information was lacking on specific drug use, comorbidities, or dietary habits, which can be potential headache-inducing factors. Moreover, data on neurological symptoms, vital signs, and other medical history were not collected. Therefore, using only the questionnaire sheets for migraine or non-migraine headache diagnoses does not allow us to ascertain their complete compatibility with clinical diagnosis by doctors. Additionally, the validity of ICHD-3 as a diagnostic tool for pediatric migraines has been a subject of discussion [97]. In clinical settings, a face-to-face consultation is obviously necessary to diagnose pediatric migraine.

Lastly, to reduce the risk of misdiagnosing potentially life-threatening conditions, a diagnostic tool must exhibit good specificity for secondary headaches. An ideal approach would involve developing an AI-based diagnostic tool that takes into account the patient’s medical history, neurological findings, and the results of radiological and laboratory tests to effectively rule out secondary headaches.

## Conclusions

This study involved the development of an AI-based diagnosis model specifically designed for pediatric and adolescent migraine using data from 909 patients. The model exhibited high diagnostic performance for identifying migraine cases accurately. The accuracy, sensitivity (recall), specificity, precision, and F-value for migraine were, respectively, 94.5%, 88.7%, 96.5%, 90.0%, and 89.4%. The ROC for migraine diagnosis, along with its AUC, was 0.99. This AI-based diagnostic tool shows promise in addressing the challenges of underdiagnosis and undertreatment of pediatric and adolescent migraine cases by non-headache specialists. However, it is essential to acknowledge certain limitations of the model, primarily due to its reliance on data obtained from questionnaire sheets. Consequently, there remains a possibility of misdiagnosis. To ensure the model’s reliability in clinical settings, further data collection and validation efforts are necessary. These steps will help enhance the model’s accuracy and usability, ultimately improving the care and management of pediatric and adolescent migraine patients.
